# Long-term tumor control following repeat gamma-knife radiosurgery of growing pituitary adenomas: a population-based cohort study

**DOI:** 10.1007/s00701-024-06341-2

**Published:** 2024-12-06

**Authors:** Felicia Lindberg, Alexander Gabri, Helena Kristiansson, Michael Gubanski, Charlotte Höybye, Martin Olsson, Petter Förander, Simon Skyrman, Bodo Lippitz, Alexander Fletcher-Sandersjöö, Jiri Bartek

**Affiliations:** 1 Department of Neurosurgery, Karolinska University Hospital, Stockholm, Sweden; 2 Department of Clinical Neuroscience, Karolinska Institute, Stockholm, Sweden; 3 Department of Endocrinology, Karolinska University Hospital, Stockholm, Sweden; 4 Department of Molecular Medicine and Surgery, Karolinska Institute, Stockholm, Sweden; 5 Department of Neurosurgery, Rigshospitalet, Copenhagen, Denmark

**Keywords:** Gamma knife radiosurgery; stereotactic radiosurgery, Pituitary adenoma, Neurosurgery, Progression-free survival, Recurrence

## Abstract

**Background:**

Gamma Knife radiosurgery (GKRS) is a well-established treatment for residual or growing pituitary adenomas (PAs) post-partial resection. However, some PAs grow even after initial GKRS, for which the efficacy of repeat GKRS is unclear. The primary objective of this study was to determine long-term progression-free survival (PFS) following repeated GKRS in patients with PA. The secondary objective was to determine predictors of tumor progression in these patients.

**Methods:**

Single-center, population-based consecutive cohort study of patients with recurrent PAs treated with repeated GKRS due to tumor progression between 1999 and 2022 at the Department of Neurosurgery, Karolinska University Hospital, Stockholm, Sweden. PFS and predictors of tumor growth were assessed.

**Results:**

23 patients were included, with a median follow-up time of 6.3 years. The 5-year PFS rate was 57%, and the median duration from repeat GKRS to tumor progression was 2.6 years. Tumor growth after repeat GKRS occurred exclusively within the first three years post-treatment. Older age at the time of repeat GKRS was a significant predictor of continued tumor growth (OR 1.09, p = 0.036).

**Conclusion:**

Repeat GKRS is a feasible treatment alternative for PAs that exhibit growth following initial GKRS.

## Introduction

Pituitary adenomas (PAs) are benign, typically slow growing, neoplasms accounting for approximately 10 – 15% of all primary intracranial tumors [11]. They can present with endocrine and/or neurological symptoms and treatment options include surgery, pharmacological treatment of hormone hypersecretion, watchful waiting [3, 13, 14], gamma-knife radiosurgery (GKRS) and fractionated radiotherapy (FRT) [4, 17, 22]. Compared to FRT, GKRS is often preferred due to its precision, reduced collateral radiation to surrounding sensitive structures, and a more rapid therapeutic effect [9]. However, tumor growth still occurs in 0–10% of PAs treated with GKRS [14, 15]. For these patients, it remains uncertain whether repeat GKRS is a viable treatment strategy.

The effectiveness of repeat GKRS for PAs that have grown after initial GKRS is documented in only a few studies with modest patient samples. Alonso et al. demonstrated in their study involving 21 patients who underwent repeat GKRS for growth hormone (GH)-secreting PAs, that 15 patients (83%) had tumor control after a median follow-up of 3.4 years; however, the indication for repeat GKRS was hormonal activity rather than tumor growth [2]. Clin et al. observed 12 patients with non-functioning PAs over a median of 7.1 years and noted tumor growth in 33% following repeat GKRS [14]. Jagannathan et al. focusing on patients with Cushing’s disease, showed that seven out of ten patients with relapse underwent repeat GKRS, with three achieving a second remission; however, the indication was relapse of Cushing’s disease, and the outcome measured was remission rather than tumor control [8]. Similarly, Mehta et al. reporting on patients with Cushing’s disease who received repeated stereotactic radiosurgery, showed a 5-year remission rate of 47% but did not present data on tumor control [16]. Additionally, a literature review on radiotherapy for refractory PAs indicated that multiple courses of radiotherapy can offer successful tumor control in specific cases [12]. Verma et al. reported a 5-year PFS rate of 58% [24]; however, the initial treatment was not specifically GKRS, and only two patients received GKRS as a second treatment, while the others received different forms of radiotherapy. Minniti et al. did a study on second course of fractionated stereotactic radiotherapy, which showed a 4-year tumor control rate of 65%; however, this study included both PAs and pituitary carcinomas and combined stereotactic radiosurgery with temozolomide [19]. Both Verma et al. and Minniti et al. suggest that repeated treatment with stereotactic radiosurgery could result in favorable outcomes for patients with growing PAs, but data on tumor control after a second GKRS remain very limited [12].

Considering the above knowledge gap, the primary aim of this study was to assess PFS following repeat GKRS in patients with PAs that exhibited growth after initial GKRS. The secondary aim was to identify possible predictors of tumor progression in these patients.

## Materials and methods

### Study design and patient selection

This was a retrospective, population-based consecutive cohort study including adult patients with recurrent PA treated with GKRS between 1999 – 2022 at the Department of Neurosurgery, Karolinska University Hospital, Stockholm, Sweden. Prior to the repeat GKRS, all patients had undergone transcranial or transsphenoidal surgical resection and a primary GKRS. The patients were identified from the Leksell Gammaplan® (Elekta Instruments Inc.) database, where all patients treated with GKRS are registered and treatment information is saved. Patients who did not reside in the Stockholm area, as well as those with insufficient or non-available follow-up data, were excluded. Patient and imaging data were collected using the health record software TakeCare ® (CompuGroup Medical Sweden AB, Farsta, Sweden). GKRS information and radiation data was collected using the Leksell Gammaplan® 10.1.1 (Elekta Instruments Inc.). The study was approved by the Swedish Ethical Review Authority (Dnr: 2017/1760–31/1), who waived the need for informed consent.

### Statistical analysis

To determine data distribution for continuous variables the Shapiro–Wilk test was used. Data is presented as median (interquartile range) for non-normally distributed data and mean ± standard deviation for normally distributed data. Categorical data is presented as numbers (proportion). The primary outcome was tumor progression, defined as MRI-verified increase in tumor size that occurred after repeat GKRS. Tumor growth was determined by a measurable increase in size on MRI compared to the previous examination, as independently assessed by two experienced neuroradiologists. To determine predictors of tumor progression, a logistic regression was performed with tumor progression as the dependent variable and possible risk factors as explanatory variables. To determine 5-year PFS, a Kaplan Meier plot was created. All analyses were performed using the statistical software R (R version 4.1.2). Statistical significance was set at p < 0.05.

### Gamma-knife treatment and follow-up

The indication for repeated GKRS was MRI-verified tumor growth of PA. In cases with tumor growth, patients were discussed at a multidisciplinary conference with the presence of radiation oncologist, neurosurgeon, neuroradiologist and endocrinologist to discuss additional treatment. Treatment option on tumor regrowth or progress included second surgery or repeat GKRS. GKRS was performed in an outpatient setting. A stereotactic frame was fixated to the skull of the patient with four screws after injection of a local anesthetic. After frame fixation, a stereotactic MRI was performed for delineation of target and adjacent structures. Based on the MRI, a treatment plan was made and GKRS was performed. After treatment, the patient stayed for monitoring in the neurosurgical ward for a few hours and was then discharged. Follow-up included MRIs at 1, 2, 5, 8 and 10 years after GKRS to find potential tumor growth, with individual variations determined by the multidisciplinary tumor board.

## Results

**Fig. 1:**
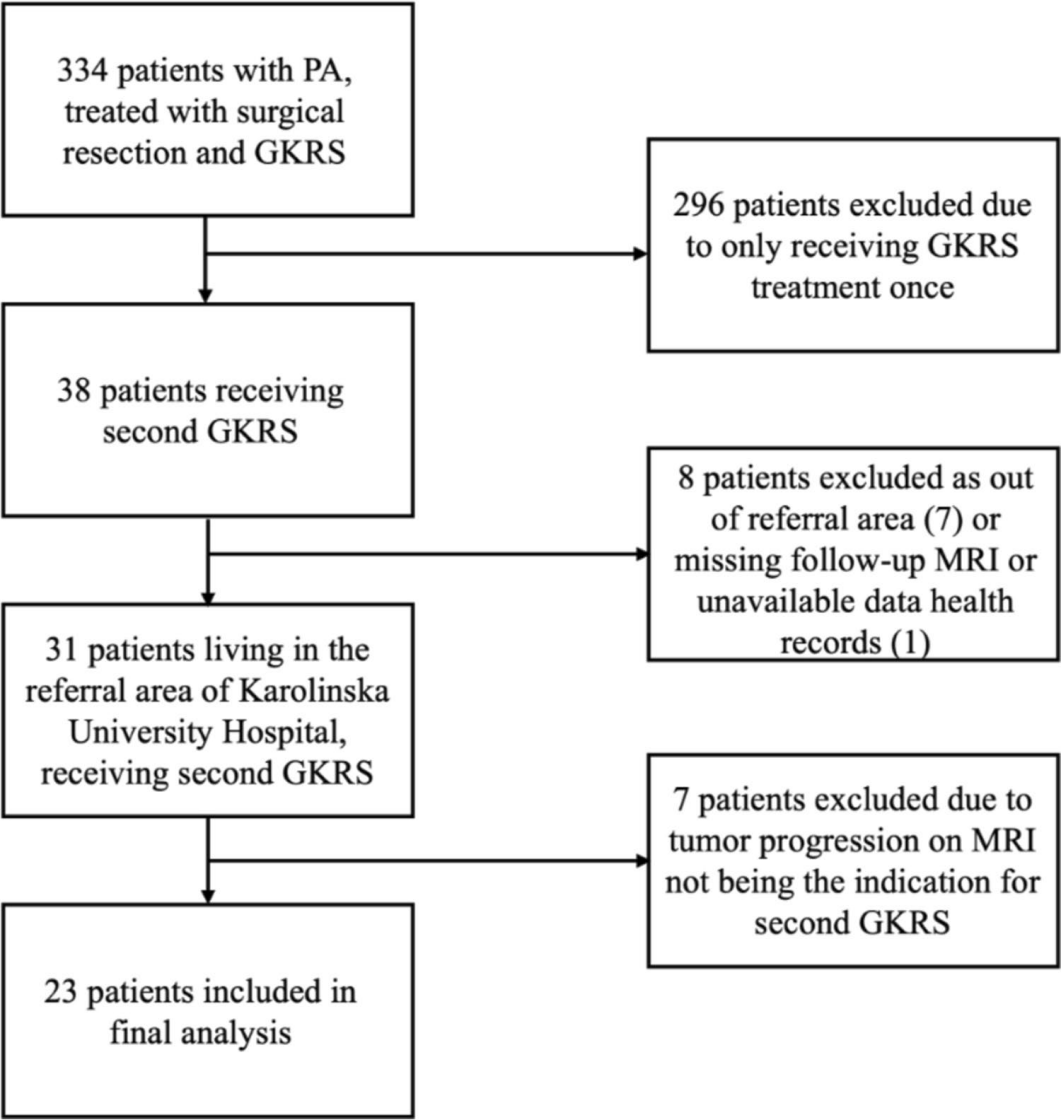
Flowchart of the patient inclusion process. Abbreviations: GKRS = Gamma-knife radiosurgery, MRI = Magnetic Resonance Imaging

A total of 334 patients with PAs treated with surgical resection and GKRS were identified. Of these, 31 were treated with repeat GKRS due to tumor growth. 8/31 patients were excluded due to residence outside of the hospital’s referral area (n = 7) or unavailable follow-up data (n = 1). Thus, 23 patients were included in the study (Fig. 1).

The cohort had a median age of 49 years at first GKRS and 57 years at repeat GKRS, and 39% were female. Median time from surgery to repeat GKRS was 6.8 years, while median time between first and the repeat GKRS was 5.2 years. 61% of PAs were NFPA, 17% ACTH-producing, 8.7% GH-producing and 13% prolactinomas. The median proliferation index (Ki67) after initial biopsy was 2% (Table 1).

**Table 1:** Descriptive data of the study cohort

Variable	All patients (n = 23)
Patient characteristics	
Female sex	9 (39%)
Age at index GKRS (years)	49 (36 – 61)
Age at repeat GKRS (years)	57 (41 – 66)
Time from first to repeat GKRS (years)	5.2 (2.9 – 7.6)
Time from surgery to repeat GKRS (years)	6.8 (4.5 – 12)
Tumor characteristics	
Tumor type	
ACTH	4 (17%)
GH	2 (8.7%)
Prolactinoma	3 (13%)
Non-functioning	14 (61%)
Proliferation Index (Ki67%)	2.0 (1.0 – 4.0) (4 missing)
Tumor volume (cm^3^) (mean (SD) and median (IQR))	1.34 (1.5686) 0.783 (0.4 – 1.8)
Treatment characteristics	
Max dose (Gy)	44 (40 – 50)
Marginal prescription dose (Gy)	22 (20 – 25)
Isodose line (%)	50 (50 – 50)
D99% (Gy)	22 (20 – 25)
Follow-up time (years)	6.3 (3.4 – 8.2)
Tumor growth after repeat GKRS	8 (35%)
Years to tumor growth	2.6 (1.8 – 3.5)
Death due to tumor	2 (8.7%)

Data presented as count (percentage), median (IQR) or mean (SD). Abbreviations: GKRS = Gamma-knife radiosurgery, IQR = Interquartile range, SD = standard deviation

The median follow-up time following repeat GKRS was 6.3 years. During this time, 8 (35%) experienced tumor growth, with a median time to growth of 2.6 years. Two patients died due to their pituitary adenomas: one died from metastasized neuroendocrine tumor (NET) originating from an atypical ACTH-producing adenoma, with the Ki-67 index increasing from 2% to over 30%; the other died from tumor burden associated with a lactotrophic pituitary NET, which had a Ki-67 index of 4%. Both cases were classified as atypical pituitary adenomas (Table 1).

In the logistic regression analysis predicting tumor growth after repeat GKRS, significant association was observed for older age (OR = 1.09, p = 0.036). No other significant predictors of tumor growth were identified (Table 2).

**Table 2:** Univariable logistic regression predicting tumor growth following repeat GKRS

Variable	OR (95% CI)	p-value
Age (years)	1.09 (1.02 – 1.21)	**0.036**
Male sex	1.11 (0.19 – 7.12)	0.907
Tumor volume (cm^3^)	2.18 (1.14 – 5.92)	0.055
Years from first to second GKRS	0.96 (0.72 – 1.23)	0.728
Proliferation index (Ki67)	1.52 (0.99 – 2.82)	0.101
Hormone secreting tumor	0.90 (0.14 – 5.23)	0.907
Follow-up time (years)	1.05 (0.86 – 1.28)	0.606

Bold text indicates a statistically significant correlation (p < 0.05). Abbreviations: GKRS = Gamma-knife radiosurgery

## Discussion

The aim of this study was to assess PFS following repeat GKRS in patients with PAs that progressed after initial GKRS. We identified a 5-year PFS of 57%, and that older age at repeat GKRS was associated with an increased risk of progression.

**Fig. 2:**
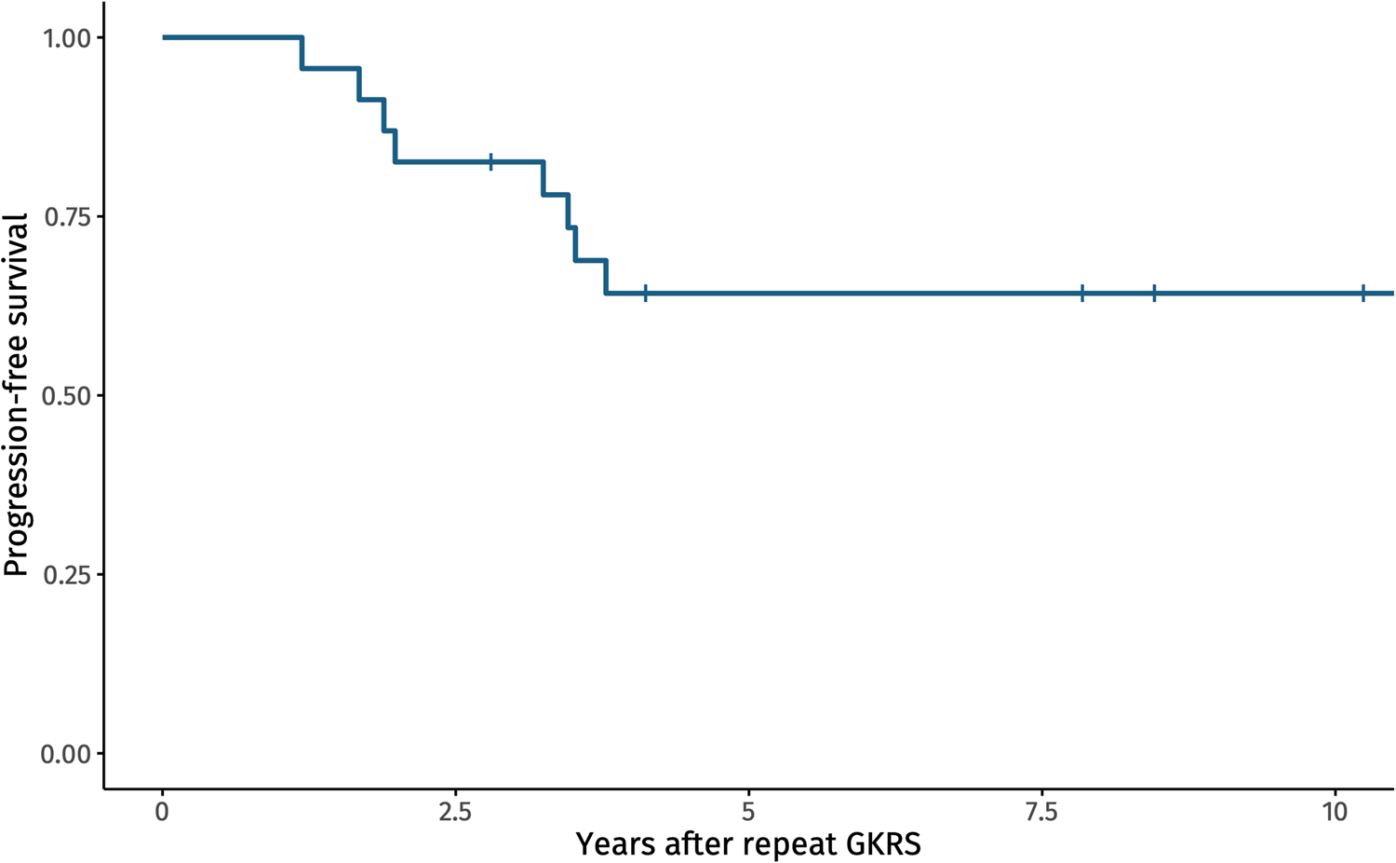
Kaplan Meier plot of tumor growth over time. 0 on the x-axis represents the time of the repeat GKRS. Each vertical line represents censoring due to stop in follow-up (last MRI)

In our study, 35% of patients experienced tumor progression following repeat GKRS, which is like the 33% progression rate reported in literature [14]. Clin et al. reporting on 12 patients, showed the median follow-up time to be 7.1 years, i.e. slightly longer than our follow-up of 6.3 years. The similarity in recurrence rates our study and Clin et al. suggests that the observed recurrence rate approximates the actual risk of progression. This inference is reinforced by Fig. 2, illustrating that if tumor progression occurs it does so exclusively within the first three years following repeat GKRS. In another study on repeated GKRS in GH secreting adenomas by Alonso et al., the progression rates were lower (16.7%) [2]. Alonso et al. did however not identify tumor growth to be the only indication for repeat GKRS, and therefore the results regarding tumor control are non-comparable [2]. Compared to data on tumor control in primary GKRS, the tumor control is lower in our study [1, 7, 20], highlighting the different biological characteristics of PAs in need of repeat GKRS.

In our cohort, older age was a significant predictor of tumor progression (OR 1.09). While no studies have reported on predictors of tumor growth specifically in the repeat GKRS setting, previous literature on primary GKRS did not identify age as a predictor; instead, tumor volume has been highlighted as a predictor of tumor growth [5, 10, 21, 23]. Older age as predictor of tumor growth, could be influenced by the fact that different pituitary adenoma types tend to occur at different ages, potentially making tumor type a confounding factor [18]. However, in our analysis, hormone secretion (used as proxy for tumor type) was not identified as a significant predictor of tumor regrowth. This suggests that age remains an independent factor in our cohort, rather than tumor type explaining the observed association. Older patients may also have reduced biological resilience, including diminished capacity for cellular repair post-radiotherapy, and may also have a higher burden of comorbidities, which could impact treatment tolerance and effectiveness. The cumulative effects of prior surgeries or radiotherapies could also influence tissue response to repeat GKRS. Moreover, it is possible that tumors in older patients may be more aggressive at the time of repeat GKRS, particularly if they have been recurrent or progressing over time. These factors may collectively explain the increased risk of tumor re-growth observed in older patients.

The discrepancy regarding tumor volume often seen as a predictor of tumor growth (while this not being the case in our study)—could at least partially be explained due to varying definitions of "large volume" across literature; prior research classified large tumors as those exceeding 5 cm^3^. Our median tumor volume was substantially smaller at 0.84 cm^3^, with a minority of patients (n = 3) having tumor volumes larger than 5 cm^3^, which may account for the observed differences in outcomes. Additionally, the frequent utilization of MRI scans in our protocol for early detection of tumor recurrence might have preempted the development of large tumors. Hence, within the context of our study, tumor volume did not emerge as a significant predictor of progression post-repeat GKRS. Corroboratively, a separate study identified that tumors under 1 cm^3^ and those not encroaching on the cavernous sinuses had a favorable response to primary GKRS, although these results are less applicable to scenarios of repeated GKRS, as they were specific to initial treatment outcomes [21].

Proliferation index is frequently cited in the literature as a prognostic factor of significance. In our analysis, the univariate logistic regression indicated an OR 1.52, with a p-value of 0.101 for the proliferation index, Ki67, treated as a continuous variable due to the lack of a defined cut-off. It's important to note that smaller sample sizes tend to result in reduced statistical power and, consequently, higher p-values. Within our cohort of 23 patients, the proliferation index was documented for 19 individuals, as Ki67 measurement was either not conducted or data was unavailable for 4 subjects. Given that our results approach statistical significance, combined with the biological rationale linking proliferation index to tumor growth [6], further investigation into the proliferation index as a prognostic factor is warranted.

### Strengths and Limitations

This study benefits from being population-based within Stockholm County, encompassing a comprehensive consecutive dataset with minimal missing data. The single-center nature of the research ensured uniformity in treatment protocols and procedures, enhancing consistency throughout the study duration and minimizing internal biases related to treatment and follow-up practices. However, it must be acknowledged that our study group is a highly selected and heterogeneous cohort, composed of patients who have undergone multiple prior treatments, indicative of challenging tumor management. The study's selective nature and the fact that these patients were deemed suitable for a repeat GKRS may affect the generalizability of the findings. Additionally, the retrospective nature of our study and the lack of a standardized volumetric cutoff for tumor re-growth should be considered. Decisions regarding re-treatment in our cohort were based on individualized clinical assessments, considering MRI findings and patient symptoms. While this reflects real-world clinical practice, it may limit reproducibility when compared to studies using rigid volumetric thresholds. Applying a strict retrospective cutoff might have failed to capture cases were timely intervention halted tumor progression, highlighting the challenge of balancing standardized criteria with individualized care. Lastly, the limited number of participants reflects the rarity of this patient group, highlighting the need for multicenter studies—preferably leveraging prospective registries—to corroborate and expand upon our findings. Despite these constraints, we believe our study contributes valuable knowledge regarding the efficacy of repeat GKRS in the management of recurrent PAs.

## Conclusion

This study demonstrated a 5-year PFS rate of 57% following repeat GKRS for growing PAs, with older age being associated with a higher risk of tumor progression. Tumor growth after repeat GKRS occurred exclusively within the first three years post-treatment. Although based on a small, selected cohort, these findings provide valuable insights into the outcomes and factors influencing the efficacy of repeat GKRS for growing PAs.

## Data Availability

Data will be made available from the corresponding author upon reasonable request.

## Abbreviations

ACTH: Adrenocorticotropic hormone
FRT: Fractionated radiotherapy
GH: Growth hormone
GKRS: Gamma knife radiosurgery
MRI: Magnetic resonance imaging
NET: Neuroendocrine cancer
NFPA: Non-functioning pituitary adenoma
PA: Pituitary adenoma
PFS: Progression free survival
